# Expression Analysis of ATP-Binding Cassette Transporters ABCB11 and ABCB4 in Primary Sclerosing Cholangitis and Variety of Pediatric and Adult Cholestatic and Noncholestatic Liver Diseases

**DOI:** 10.1155/2019/1085717

**Published:** 2019-12-10

**Authors:** Cornelia Thoeni, Ruediger Waldherr, Jutta Scheuerer, Stefanie Schmitteckert, Ralph Roeth, Beate Niesler, Ernest Cutz, Christa Flechtenmacher, Benjamin Goeppert, Peter Schirmacher, Felix Lasitschka

**Affiliations:** ^1^Institute of Pathology, University Hospital Heidelberg, Heidelberg, Germany; ^2^Division of Laboratory Medicine and Pathobiology, University of Toronto, Toronto, Ontario, Canada; ^3^Department of Human Molecular Genetics, Institute of Human Genetics, University Heidelberg, Heidelberg, Germany; ^4^nCounter Core Facility Heidelberg, Exzellenzcluster CellNetworks, University Heidelberg, Heidelberg, Germany; ^5^Division of Pathology, Department of Paediatric Laboratory Medicine (DPLM), The Hospital for Sick Children, University of Toronto, Toronto, Canada; ^6^Institute of Pathology, Ludwigshafen, Germany

## Abstract

ATP-binding cassette (ABC) transporters are the members of the efflux pumps that are responsible for the removal of cytotoxic substances by active transport. ABCB11, the bile salt efflux pump of hepatocytes, coordinates cellular excretion of numerous conjugated bile salts into the bile canaliculi, whereas ABCB4 acts as an ATP-dependent floppase translocating phosphatidylcholine from the inner to the outer leaflet of the bile canalicular membrane. Loss of functional ABCB11 and ABCB4 proteins causes early-onset refractory cholestasis or cholangiopathy. In this study, we investigated the expression and localization pattern of ABCB11 and ABCB4 using immunohistochemistry and RNA profiling in liver samples from patients with different types and stages of chronic cholestatic liver disease, with emphasis on primary sclerosing cholangitis (PSC), compared to a variety of cholestatic and noncholestatic hepatopathies. Therefore, ABCB11 and ABCB4 expressions were investigated on formalin-fixed and paraffin-embedded (FFPE) material in a patient cohort of total 43 patients with or without cholestatic liver diseases, on protein level using immunohistochemistry and on RNA level using nanoString technology. Intriguingly, our results demonstrated increased expression of ABCB11 and ABCB4 on protein as well as RNA level in PSC, and the expression pattern correlated with disease progression. We concluded from our study that patients with PSC demonstrate altered expression levels and pattern of ABCB11 and ABCB4 which correlated with disease progression; thereby, ABCB11 and ABCB4 analysis may be a useful tool for assessment of disease stages in PSC.

## 1. Introduction

In normal hepatocytes, the bile release occurs at the canalicular membrane predominantly through the action of transporters belonging to the adenosine triphosphate-binding-cassette (ABC) family [1, 2]. ABCB11 and ABCB4 are the two principal transporters of this family. ABCB11 is the main bile salt transporter in hepatocytes that is expressed at the apical membrane of hepatocytes, and when dysfunctional leads to various cholestatic disorders including intrahepatic cholestasis in pregnancy (ICP), progressive familiar intrahepatic cholestasis (PFIC), and transient neonatal cholestasis [1, 3–6].

ABCB4 is also expressed at the apical membrane of hepatocytes and is essential for phosphatidylcholine secretion into the bile. Defective function of ABCB4 causes imbalance in the composition of primary bile, with lack of phosphatidylcholine and an overload of bile salts. As previously shown, in PFIC, a dysfunctional ABCB4 protein leads to damage of bile canaliculi and small bile ducts and causes chronic and progressive liver disease [1–6]. It has been suggested recently that genetic variants of ABCB11 and ABCB4 could be involved in the pathogenesis of primary sclerosing cholangitis (PSC) [7, 8]. However, the precise mechanisms are unknown [9–12]. In general, PSC is a rare disorder characterized by chronic intra- and extrahepatic bile duct inflammation leading to progressive periductal fibrosis, multifocal bile duct strictures, dilatation (cholangiectasis), progressive cholestatic liver disease and hepatic dysfunction [11–13].

The aim of this study was to investigate the expression and localization of the critical bile transporters ABCB11 and ABCB4 in liver tissue samples from patients with different types of chronic cholestatic and noncholestatic liver disease, with particular emphasis on PSC. Here we show that, in PSC patients, ABCB11 and ABCB4 are significantly overexpressed when compared to other cholestatic and noncholestatic liver diseases. Moreover, in PSC patients, a strong correlation exists between their expression and localization as well as the grade of cholestasis and disease progression. These findings indicate that, in patients with PSC, chronic cholestasis leads to compensatory upregulation of critical ATP-binding cassette (ABC) transporters in hepatocytes. Thereby, immunohistochemistry for ABCB11 and ABCB4 could be a useful diagnostic tool to determine the extent of cholestasis and disease progression in PSC.

## 2. Materials and Methods

### 2.1. Patient Cohort and Tissue Material

The patient cohort of this study consisted of 43 patients in total, of whom either liver explants, resections, or liver biopsies were available. The patients were selected on the basis of following criteria: (1) sufficient material for liver microscopy, immunohistochemistry, and molecular biology and (2) definite diagnosis based on clinical, laboratory, and morphological data. Therefore, 28 patients with other liver diseases than PSC were compared to 15 PSC patients. Most PSC patients presented also with ulcerative colitis (Supplementary [Supplementary-material supplementary-material-1]).

The non-PSC cohort was grouped into 2 main categories: (1) pediatric chronic liver diseases (most of those patients were diagnosed with progressive familial intrahepatic cholestasis, PFIC) and (2) adult chronic liver diseases other than PSC.

The pediatric chronic liver disease group consists of total 17 patients with neonatal cholestasis (including 3 patients with Alagille syndrome), one patient with short bowel syndrome and liver disease related to TPN (total parenteral nutrition), and 13 patients with a diagnosis of definite PFIC based on genetic sequencing (types 1–3, *n*=10), or showed clinical lab parameters as markedly elevated gamma GT and histological hallmarks for cholestatic liver disease, to be suspicious for the diagnosis of PFIC, but without evidence of a genetic mutation, as sequencing analysis data were not available. However, from two patients with progressive familial intrahepatic cholestasis type 3 (PFIC3), genetic sequencing data were available. Those PFIC3 patients were siblings. In both patients, a specific homozygous mutation in the ABCB4 gene (p.H1238Y) was identified which was previously described to cause PFIC3 in the liver (6).

The adult chronic liver disease group consists of total 11 patients, 5 patients with chronic hepatitis C, 3 patients with NASH (nonalcoholic steatohepatitis), 2 patients with FNH (focal nodular hyperplasia), and 1 patient with PCLD (polycystic liver disease). In the patient with PCLD as well as the patients with FNH, nonaffected, lesion-free liver tissue was investigated.

Liver disease progression and staging including cholestasis and fibrosis grade were assessed by total serum bilirubin, as a clinical lab parameter, as well as by histology. For histological evaluation of liver disease and disease progression, liver sections were evaluated with the Nakanuma Scoring and Staging system, which was introduced in 2010 to define the following 3 features: fibrosis, bile duct loss as well as cholestasis grade, finally assessing progression of cholestatic liver disease in PBC patients [14, 15]. Clinical parameters (type of disease, tissue type, sex, age and total bilirubin, and presence of ulcerative colitis in PSC patients) are summarized in Supplementary [Supplementary-material supplementary-material-1], and histology parameters (Nakanuma Score and Stage) and assessment of ABCB11 and ABCB4 expression patterns are summarized in Supplementary [Supplementary-material supplementary-material-1].

### 2.2. Immunohistochemistry

FFPE liver specimens of all 43 patients between 2000 and 2015 were retrieved from the archive of the Institute of Pathology, University Hospital Heidelberg, Ruprecht-Karls-University Heidelberg, Germany, with the support and under the regulation of the NCT Tissue Biobank. Liver tissues were used in accordance with the ethical guidelines of the NCT Tissue Biobank as defined by the local ethics committee (ethical vote 206/05).

In brief, liver sections were cut at 2-3 *μ*m thickness, deparaffinized with xylene, and afterwards rehydrated using graded alcohols. Immunofluorescence for Peroxisomal Biogenesis Factor 11 Beta (PEX11B) was performed according to previously published protocols [16].

Antigen retrieval for immunohistochemistry was performed with EDTA buffer, pH 9, and high-pressure cooking for 5 minutes. Blocking was performed with 15% goat serum (Vector, Germany) and antibody diluent solution (Life Technologies, Germany) for 1 hour at room temperature. Primary antibody incubation was performed at 4°C overnight.

Next day, sections were washed 3 times with 1X Tris-buffered saline, 0.1% Tween® for 10 minutes and incubated light protected with secondary antibodies. Afterwards, sections were subsequently washed light protected 3 times for 10 minutes and incubated with DAB solution (ImmPACT™ DAB, Peroxidase Substrate Kit, Linaris, Germany) for 1 minute. Nuclei were counterstained with Mayer's Hematoxylin in immunohistochemistry. Hoechst 33342 staining solution (Thermo Fisher Scientific, Germany) was used to label nuclei in immunofluorescence.

Finally, sections were mounted using Aquatex® Mounting Medium (Merck-Millipore, Germany) or ProLong™ Diamond Antifade Mountant solution (Life Technologies, Germany).

Primary and secondary antibodies used for immunohistochemistry/immunofluorescence are summarized in Supplementary [Supplementary-material supplementary-material-1]. Brightfield images of the immunohistochemical staining were taken with an Olympus Microscope BX53 equipped with an Olympus Camera SC30, images of immunofluorescence stained sections with an Olympus microscope equipped with a Leica DFC365FX camera. Subsequently, images were adjusted for brightness and contrast using the Photoshop CS5 software.

### 2.3. Shikata's Orcein Staining of FFPE Liver Tissue

For evaluation of cholestasis degree, liver sections were stained with Shikata`s orcein staining kit (Clin Tech Limited, UK) according to previously published protocols [14, 15]. The orcein stain is used to identify copper binding protein that is increased in chronic cholestasis. In brief, liver sections were cut at 3 *μ*m thickness, deparaffinized with xylene, rehydrated using graded alcohols, and washed 3 times with distilled water. Next, liver sections were oxidized light protected in 0.5% potassium permanganate for 5 minutes and in 1% oxalic acid for 2 minutes. Afterwards, liver sections were rinsed in running tap water, washed 3 times with distilled water, dipped several times in 70% ethanol, and stained with Shikata's Orcein for 1 minute. Finally, liver sections were washed with distilled water and 70% ethanol, dehydrated in graded alcohols, cleared in xylene, and mounted in Consul-Mount™ mounting medium (Thermo Fisher Scientific, Germany). Brightfield images were taken with an Olympus Microscope BX53 equipped with an Olympus Camera SC30 and adjusted for brightness and contrast using Photoshop CS5 software.

### 2.4. Laser Capture Microscopy (LCM) and RNA Extraction

LCM was performed to specifically isolate hepatocytes from FFPE liver explant and resection samples of 10 PSC patients, 8 patients with chronic liver diseases other than PSC (FNH, chronic hepatitis C and PCLD), and 2 siblings from the neonatal cholestasis group, where a specific disease-causing mutation in the ABCB4 gene was identified to cause PFIC3 (illustrated in Supplementary [Supplementary-material supplementary-material-1]).

In brief, 10 *μ*m thick sections of FFPE liver explants were cut and deposited on PEN membrane slides (Leica Microsystems, Germany), dried, deparaffinized with xylene, and rehydrated with graded concentrations of ethanol. LCM was performed using a Leica LMD 7000 (Leica Microsystems, Germany) as previously described [17]. RNA extraction was performed using the High Pure FFPET RNA Isolation Kit according to the manufacturer's instructions (Roche, Germany).

### 2.5. nCounter Gene Expression Profiling on LCM Samples from FFPE Liver Specimen Sections

In total, 20 LCM-FFPE liver explant samples or resections were used for nCounter gene expression profiling (nanoString, Seattle), including LCM-FFPE liver tissue of 10 PSC patients presenting with cholestatic liver disease (disease group), 8 patients with chronic liver diseases other than PSC (control group), such as FNH, chronic hepatitis C, and PCLD, and two patients with PFIC3 (summarized in Supplementary [Supplementary-material supplementary-material-1]). In the patient with PCLD as well as the patients with FNH, nonaffected, lesion-free liver tissue was extracted and investigated.

In brief, 50 ng of total RNA from LCM isolated FFPE liver explant samples was used to assess ABCB11 and ABCB4 RNA expression profiles by nCounter analysis at the nCounter Core Facility Heidelberg using the nCounter SPRINT system and nCounter Elements chemistry according to the manufacturer's instructions. Detailed probe design for the ABCB11 and ABCB4 genes as well as for the reference genes is given in Supplementary [Supplementary-material supplementary-material-1]. For determination of the most stably expressed reference genes, the geNORM method was applied. In total, four stably expressed reference genes *UBC*, *GAPDH*, *ACTB*, and *PEX11B* were used for the comparative analysis of differences in ABCB11 and ABCB4 RNA expression between patient and control samples. Out of the four housekeeping genes, *PEX11B* was the gene, which showed the most equal expression in all patient populations. Therefore, ABCB11 and ABCB4 RNA expression was compared and normalized to *PEX11B* expression in all patients. Background correction and normalization of data were performed with the nSolver Analysis Software 3.0 (nanoString Technologies, Seattle). Gene expression values are presented as CodeSet counts ± SD. Statistical analysis was performed with the statistical software GraphPadPrism Version 8.0d using the unpaired *t* test (two-tailed). Samples with a *p* value ^*∗*^*p* < 0.05 were considered as statistically significant (^*∗*^*p* < 0.05, ^*∗∗*^*p* < 0.01, ^*∗∗∗*^*p* < 0.001, and ^*∗∗∗∗*^*p* < 0.0001). Data obtained from the nanoString expression profile of FFPE liver explants were validated on protein expression levels by immunohistochemistry for ABCB11 and ABCB4 as well as immunofluorescence for PEX11B (reference gene).

## 3. Results

### 3.1. Clinical and Histologically Evaluation of Chronic Cholestatic Liver Disease

All patients were evaluated for cholestasis grade clinically with total serum bilirubin and histologically with the Nakanuma Scoring and Staging system [14, 15], summarized in Supplementary Tables [Supplementary-material supplementary-material-1] and [Supplementary-material supplementary-material-1].

All PSC patients demonstrated significant elevated total serum bilirubin levels and most PSC patients, in particular patients who underwent liver transplantation and where the liver explant was investigated, presented with an advanced disease stage (Nakanuma 3/4, Supplementary Tables [Supplementary-material supplementary-material-1] and [Supplementary-material supplementary-material-1]). Total bilirubin levels correlated with histological disease stage, patients with Nakanuma stage 3/4 showed significant increased total bilirubin levels (>3 mg/dl) compared to patients with Nakanuma stage 2 (Supplementary Tables [Supplementary-material supplementary-material-1] and [Supplementary-material supplementary-material-1]). Also, the PSC group was the only group where all patients showed histologically orcein-positive granules, indicating an increased amount of copper-binding protein in the cytoplasm of hepatocytes as a sign of long-lasting cholestasis (Supplementary [Supplementary-material supplementary-material-1]).

In the pediatric chronic liver disease group, patients with Alagille syndrome showed significant higher total bilirubin serum levels (>5 mg/dl) as well as more advanced histological disease stage in the initial biopsy or the liver explant when undergoing transplantation (Nakanuma 3/4) compared to patients with PFIC or the patient with cholestasis related to TPN (Supplementary Tables [Supplementary-material supplementary-material-1] and [Supplementary-material supplementary-material-1]). Most PFIC patients showed on the initial biopsy Nakanuma stage 2 and in the liver explant Nakanuma stage 3 (Supplementary [Supplementary-material supplementary-material-1]).

Finally, from the adult chronic liver disease group other than PSC, most patients have chronic hepatitis C. Patients with hepatitis C and low serum bilirubin levels (total serum bilirubin levels <2 mg/dl) showed histologically a Nakanuma stage 2, and patients with chronic hepatitis C and advanced disease progression (Nakanuma stage 3) presented with higher total serum bilirubin levels (>2 mg/dl).

Patients with NASH and FNH and the patient with PCLD showed total serum bilirubin levels (<1 mg/dl). FNH and the patient with PCLD were the only patients with Nakanuma stage 1; however, NASH patients demonstrated Nakanuma stage 2, as of advanced fibrosis (Supplementary [Supplementary-material supplementary-material-1]).

### 3.2. Immunohistochemistry Analysis of ABCB11 and ABCB4 in Chronic Cholestatic Liver Disease

Expression and localization of the ATP-binding cassette (ABC) transporters ABCB11 and ABCB4 in hepatocytes were investigated in all 43 patients. Thereby, different expression and localization patterns of the ATP-binding cassette (ABC) transporters ABCB11 and ABCB4 were observed.

**Figure 1:**
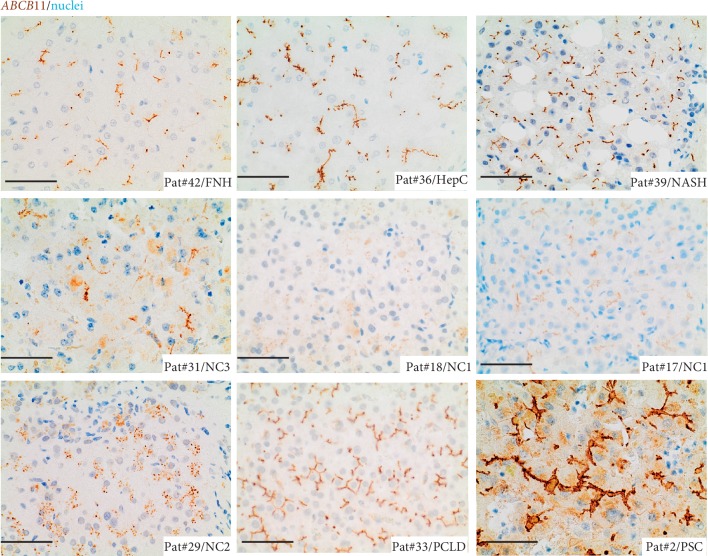
Immunohistochemical staining for ABCB11 in FFPE liver sections of FNH, chronic hepatitis C, NASH, Alagille syndrome (NC3), PFIC (NC1), TPN-related liver disease (NC2), PCLD, and PSC. ABCB11 stained in brown; nuclei stained with hematoxylin in blue. In PSC, high expression and accumulation of ABCB11 at the bile canalicular membrane of adjacent hepatocytes were most consistent between different cholestatic liver diseases. Scale bar = 20 *μ*m.

**Figure 2:**
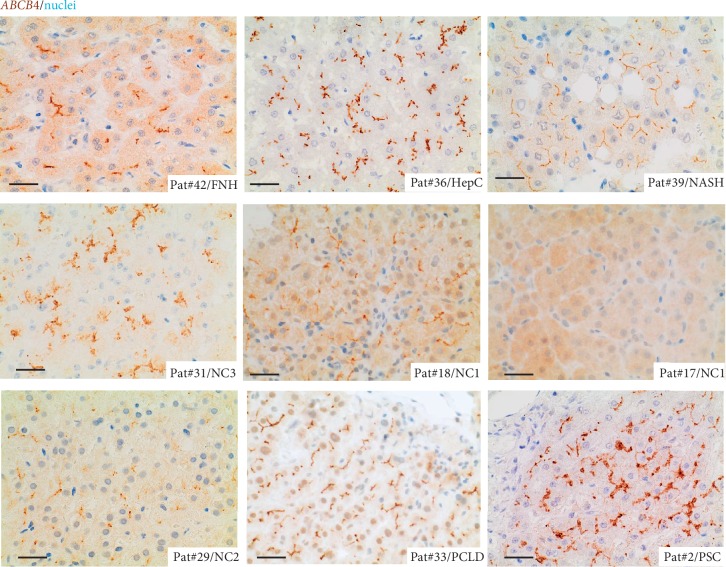
Immunohistochemical staining for ABCB4 in FFPE liver sections of FNH, chronic hepatitis C, NASH, Alagille syndrome (NC3), PFIC (NC1), TPN-related liver disease (NC2), PCLD, and PSC. ABCB4 stained in brown; nuclei stained with hematoxylin in blue. In PSC, high expression and accumulation of ABCB4 at the bile canalicular membrane of adjacent hepatocytes were mostly consistent within the group of cholestatic liver diseases. Scale bar = 20 *μ*m.

Patients with NASH, as well as nonaffected, lesion-free liver tissue of the PCLD patient, demonstrated homogeneous expression of both transporters at the bile canalicular membrane of adjacent hepatocytes (Figures 1 and 2; Supplementary [Supplementary-material supplementary-material-1]). In contrast, patients with chronic liver disease related to chronic hepatitis C, and pediatric/neonatal cholestasis, including Alagille, PFIC, and TPN demonstrated a broad spectrum of expression levels and subcellular localization (Figures 1 and 2, Supplementary [Supplementary-material supplementary-material-1] and Supplementary [Supplementary-material supplementary-material-1]). In particular, patients with progressive familial intrahepatic cholestasis (PFIC) type 2 and type 3 showed a broad variety in ABCB11 and ABCB4 staining in representative disease sections, which might be best explained by the underlying mutations within the ABCB11 and ABCB4 genes and therefore dependent on the effect of the disease-causing mutation on protein function, as previously described (6). PFIC2 patients with mutations in the ABCB11 gene presented with either a decreased expression of the ABCB11 at the bile canaliculi or cytoplasmic mislocalization of ABCB11, or both (Figure 1 and Supplementary [Supplementary-material supplementary-material-1]). ABCB4 staining in PFIC2 patients was either homogeneous at the bile canaliculi or inhomogeneous with weak expression or accumulation of ABCB4 at the bile canalicular membrane (Figure 2 and Supplementary [Supplementary-material supplementary-material-1]). Next, PFIC3 patients presented either with a homogeneous or inhomogeneous staining pattern of ABCB11 with weak expression, accumulation, or cytoplasmic mislocalization of ABCB11 in hepatocytes (Figure 1 and Supplementary [Supplementary-material supplementary-material-1]). A same staining pattern was observed for ABCB4, except that the staining pattern was inhomogeneous (Figure 2 and Supplementary [Supplementary-material supplementary-material-1]). Interestingly, in two siblings with a specific disease-causing mutation in the ABCB4 gene (p.H1238Y), different staining patterns for ABCB11 and ABCB4 were observed in different areas of cholestatic liver disease (Supplementary [Supplementary-material supplementary-material-1], Supplementary [Supplementary-material supplementary-material-1]). In both PFIC3 patients, different areas of cholestatic liver parenchyma were investigated for ABCB11 and ABCB4 protein expression in immunohistochemistry (Supplementary [Supplementary-material supplementary-material-1] and Supplementary [Supplementary-material supplementary-material-1]). Both transporters demonstrated areas with either weak expression or strong/accumulation at the bile canalicular membrane with occasional cytoplasmic mislocalization of both transporters (Supplementary [Supplementary-material supplementary-material-1] and Supplementary [Supplementary-material supplementary-material-1]).

One PFIC1 patient showed marked cytoplasmic mislocalization of ABCB11, whereas ABCB4 accumulated at the bile canalicular membrane; the second patient was clinically/histologically suspicious for a PFIC1 diagnosis, but no genetic sequencing analysis data were available; the patient showed homogeneous expression of both transporters at the bile canalicular membrane (Supplementary [Supplementary-material supplementary-material-1] and Supplementary [Supplementary-material supplementary-material-1]).

Alagille patients presented mostly with accumulation of ABCB11 and ABCB4 at the bile canalicular membrane of adjacent hepatocytes, whereas the patient with total parenteral nutrition (TPN) showed homogeneous cytoplasmic mislocalization of ABCB11 and inhomogeneous, weak expression or areas of accumulation of ABCB4 at the bile canalicular membrane (Figures 1 and 2, Supplementary [Supplementary-material supplementary-material-1]).

Patients from the adult chronic liver disease group other than PSC showed mostly a homogeneous staining pattern for both transporters (Figures 1 and 2, Supplementary [Supplementary-material supplementary-material-1], and Supplementary [Supplementary-material supplementary-material-1]). One patient with chronic hepatitis C showed inhomogeneous, weak expression of ABCB11 and ABCB4, and one FNH patient, accumulation of ABCB4 in some areas, but a homogeneous expression of ABCB11 at the bile canalicular membrane of adjacent hepatocytes (Supplementary [Supplementary-material supplementary-material-1] and Supplementary [Supplementary-material supplementary-material-1]).

In contrast, the staining pattern of ABCB11 and ABCB4 was mostly consistent in all PSC patients, when compared to patients with other chronic liver/cholestatic liver diseases. ABCB11 and ABCB4 were strongly expressed and accumulated at the bile canalicular membrane of adjacent hepatocytes in PSC patients (Figures 1 and 2, Supplementary [Supplementary-material supplementary-material-1], and Supplementary Figures [Supplementary-material supplementary-material-1] and [Supplementary-material supplementary-material-1]).

**Figure 3:**
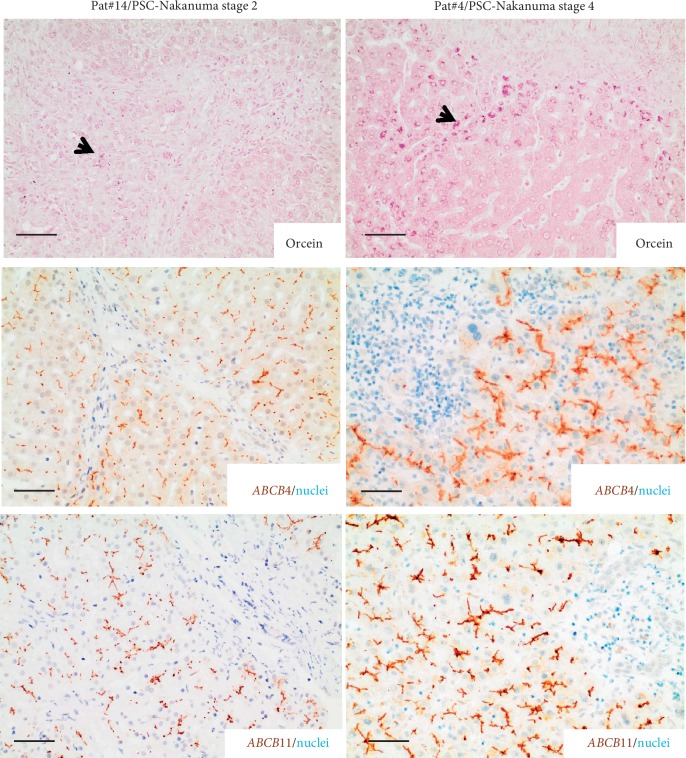
Shikata's orcein and immunohistochemical staining for ABCB4 and ABCB11 in FFPE liver biopsies and explants of PSC patients with different stages of chronic cholestatic liver disease. Orcein granules are stained dark pink in hepatocytes (black arrows). ABCB4 and ABCB11 stained in brown; nuclei stained with hematoxylin in blue. PSC patients with mild disease progression (Nakanuma stage 2) demonstrate focally areas of periportal (zone 1) hepatocytes with strong ABCB4 and ABCB11 expressions at the bile canalicular membrane, whereas PSC patients with advanced disease progression and extensive upregulation of ABCB4 and ABCB11 expression were seen at the bile canalicular membrane of mostly all hepatocytes (zones 1–3). Scale bar = 20 *μ*m.

Interestingly, the correlation between cholestatic liver disease stage and expression pattern of both ATP-binding cassette (ABC) transporters was observed in PSC. Thus, biopsies of PSC patients with mild cholestatic liver disease (Nakanuma stage 2), strong ABCB11, and ABCB4 expression/accumulation at the bile canalicular membrane were only present focally in some periportal hepatocytes (zone 1 hepatocytes, Figure 3 and Supplementary [Supplementary-material supplementary-material-1]); in contrast, in liver tissue of PSC patients with advanced cholestatic liver disease (Nakanuma stage 3-4), ABCB11 and ABCB4 were overall strongly expressed and not only focally in periportal hepatocytes (zone 1 hepatocytes), but also in areas around the central vein (zone 2/3 hepatocytes, Figure 3 and Supplementary [Supplementary-material supplementary-material-1]).

### 3.3. RNA Expression Analysis of ABCB11 and ABCB4 in Chronic Cholestatic Liver Disease

**Figure 4:**
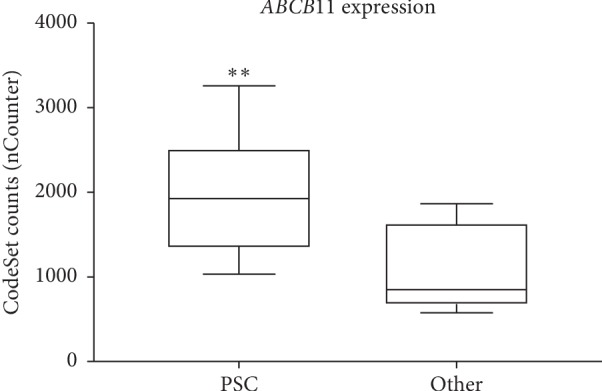
Comparative expression analysis of RNA levels of ABCB11 in LCM hepatocytes of FFPE liver explants from PSC patients (PSC, *n*=10) and patients with non-PSC liver diseases (Other, *n*=8). nCounter analysis revealed significantly differential expressed RNA levels of the ABCB11 gene between the 2 patient cohorts. ABCB11 is higher expressed in hepatocytes of PSC patients when compared to the control group (Other). *p* values were determined by an unpaired *t* test (two-tailed) (^*∗∗*^*p* < 0.0061).

**Figure 5:**
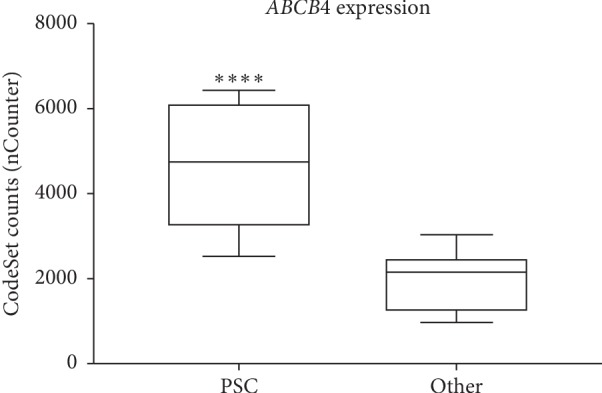
Comparative expression analysis of RNA levels of ABCB4 in LCM hepatocytes of FFPE liver explants from PSC patients (PSC, *n*=10) and patients with non-PSC liver diseases (Other, *n*=8). nCounter analysis revealed significantly differential expressed RNA levels of the ABCB4 gene between the 2 patient cohorts. ABCB4 is higher expressed in hepatocytes of PSC patients when compared to the control group (Other). *p* values were determined by an unpaired *t* test (two-tailed) (^*∗∗∗∗*^*p* < 0.0001).

In addition, complementing RNA profiling using the nCounter nanoString technology was performed on LCM dissected FFPE liver explant samples from 10 PSC patients with cholestatic liver disease (disease group), 8 patients without PSC (control group), as well as 2 siblings from the pediatric/neonatal cholestatic liver disease group with a homozygous mutation in the ABCB4 gene (p.H1238Y). RNA of both transporters, ABCB11 and ABCB4 were significantly higher expressed (^*∗∗*^*p* < 0.0061 for ABCB11 and ^*∗∗∗∗*^*p* < 0.0001 for ABCB4) in PSC patients as well as both PFIC3 patients when compared to patients without PSC disease (Figures 4 and 5, Supplementary Figures [Supplementary-material supplementary-material-1] and [Supplementary-material supplementary-material-1] and Supplementary [Supplementary-material supplementary-material-1]). However, correlation between upregulated RNA and homogeneous increased protein expression was only consistent in the PSC group, not within the PFIC3 group.

In conclusion, PSC patients showed an increased expression of RNA and protein of ABCB11 and ABCB4 in hepatocytes, expression correlated with disease stage and progression, and the expression pattern of both transporters was mostly consistent in PSC patients when compared to other cholestatic liver diseases.

## 4. Discussion

Cholestasis is characterized by intrahepatic accumulation of potentially cytotoxic bile acids leading to liver injury, disruption of hepatocellular integrity, fibrosis, and eventually liver cirrhosis [18–20]. Bile salt export pump (ABCB11) as well as the multidrug-resistant P-glycoprotein 3 transporter (ABCB4) are critical proteins to coordinate the detoxification process by removing bile compounds from hepatocytes [18–20]. Absence of ABCB11 and ABCB4 proteins related to mutations in the ABCB11 and ABCB4 gene causes PFIC2 and 3 and leads to severe cholestatic liver disease in children that results in development of progressive cholestasis and liver cirrhosis [1–6, 18–20]. Functional *in vivo* analysis in rodents has been performed for both transporters to investigate in detail the effect of loss of ABCB11 and ABCB4 in chronic cholangiopathies and chronic cholestatic liver diseases [18–20]. In contrast to patients with cholestatic liver disease related to ABCB11 mutations, functional analysis of ABCB11 deficiency in rodents showed that ABCB11 deficient mice did not develop progressive cholestasis and were associated with a milder phenotype when compared to *MDR2* deficient mice [18–21]. Megaraj et al. published a study where ABCB11-deficient mice showed efficient synthesis of specific bile acids, tetrahydroxy bile acids [22]. Those bile acids seemed to be mediated through different bile transporter pathways, Mrp2 and P-gp substrates [22]. Mediation through different bile transport pathways explained the noncholestatic phenotype in ABCB11 deficient mice [22]. The concept of bypassing bile export from the hepatocytes with activation of other bile transporters might be also considered in PSC patients. Moreover, recently, it has been reported that overexpression of hepatic ABCB11 in mice increased the conservation of primary bile acids (cholic acid) within the enterohepatic circulation, which highly suggested the existence of a feed-forward communication between expression of a bile acid transport protein as ABCB11 and the intestine [23]. This is an interesting concept and might give another explanation why PSC patients in our study showed increased ABCB11 RNA and protein expression, as those patients often present with elevated bile acids in the bloodstream, as a sign for altered enterohepatic circulation.

Interestingly, mice deficient in the canalicular phospholipids floppase (*MDR2*, the murine orthologue of human ABCB4) not only serve as a model for functional *in vivo* analysis of human ABCB4 deficiency but also as a model for chronic biliary liver diseases as PSC [18, 24]. Those animals spontaneously develop pericholangitis, ductular proliferation, and typical onion-skin type periductal fibrosis as a result of defective biliary phospholipid secretion and subsequent increase of free nonmicellar and toxic bile acid concentrations in the bile [18, 24]. Development of periportal biliary fibrosis in ABCB4 deficient animals might be caused by toxic bile leakage into portal tracts by periportal and/or peribiliary myofibroblasts [18–20, 24]. Moreover, heterozygous mutations in the ABCB4 gene have been described in patients with ICP as well as adults with unexplained anicteric cholestasis and pronounced liver fibrosis [19]. In those patients, ABCB4 was significantly reduced in the bile canalicular membrane [19]. As ABCB11 and ABCB4 deficiencies in mice, in particular ABCB4 deficiency, are associated with fibrotic biliary disease, genetic variations have been discussed in the pathogenesis of PSC as well. Indeed, intracellular accumulation of toxic bile constituents and altered bile composition resulting in cellular damage and cell death have been hypothesized to contribute to the pathogenesis of PSC [9–14, 18]. But so far, neither disease causing mutations nor alterations in expression patterns of those transporters has been described in patients with PSC. Therefore, we investigated in this study ABCB11 and ABCB4 on RNA as well as protein levels in liver tissue of patients with different types of cholestatic liver diseases, with a special interest in PSC.

We could show in all patients with PSC that both transporters, in particular ABCB4, were uniformly strongly expressed and accumulated at the bile canalicular membrane. Overexpression was also confirmed on RNA levels of both transporters, especially the ABCB4, implicating that chronic cholestatic disease may lead to upregulation of ABCB11, and more evident ABCB4, potentially as a compensatory mechanism to remove the overload of toxic bile constituents, which accumulated during period of long-lasting chronic cholestasis in PSC. This phenomenon was supported, as in PSC, ABCB11, and ABCB4 expression correlated with disease stage as well as disease progression. Thus, PSC patients with early disease and mild disease progression (Nakanuma stage (2) demonstrated focal overexpression and accumulation of ABCB11 and ABCB4 at the bile canalicular membrane of periportal hepatocytes (zone 1 hepatocytes), whereas in PSC patients with moderate to severe disease progression (Nakanuma stage 3-4), ABCB11 as well as ABCB4 expression were highly overexpressed in hepatocytes of zone 1, 2, and 3 and not restricted to periportal hepatocytes (zone 1 hepatocytes).

Interestingly, the PSC cohort showed the most consistent expression pattern of ABCB11 and ABCB4, and the protein expression pattern correlated with RNA expression. This was not the case in the two siblings with PFIC3, who also presented with increased RNA but showed different grades of protein expression of both transporters in different areas of representative cholestatic liver disease sections in immunohistochemistry, which might be related to the specific mutation (p.H1238Y) within the ABCB4 gene [6].

Megaraj et al. described nicely the coordination through other bile export transporting pathways if one bile transporter is defect. This could also be the case in PSC patients, as not only ABCB11, but also ABCB4 was upregulated. We also measured RNA expression of the ATP8B1 (FIC1), a transporter, which is mutated in PFIC1 patients [1, 3, 5, 6]. Interestingly, ATP8B1 was expressed equally in all 3 cholestatic liver disease groups (PSC, adult cholestatic liver disease other than PSC, and the PFIC3 group, data not shown). Therefore, ABCB11 and ABCB4 upregulation in PSC patients might be specific. However, investigating other transporters such as the MRP2, a transporter mutated in patients with Dubin-Johnson syndrome [1, 3, 5, 6], or the Farnesoid X receptor, a transporter triggering hepatic inflammation via the NF-*κ*B pathway [25], would be helpful in future studies on a larger population of PSC patients to further elucidate the disease mechanisms of cholestasis development due to altered expression of transporters in hepatocytes of patients with chronic cholestatic liver diseases as PSC.

## 5. Conclusion

In summary, our study demonstrates that, in patients with PSC, chronic cholestasis leads to upregulation of ABCB11 and ABCB4 expressions at RNA and protein levels in hepatocytes, which is likely to be a compensatory mechanism to counteract the accumulation of toxic bile constituents. Furthermore, upregulation and increased expression of these transporters correlated with cholestatic liver disease stage and disease progression in PSC. Increased expression of ABCB11 and ABCB4 was very consistent in PSC patients and not observed in other cholestatic liver diseases. Thereby, immunohistochemical assessment of ABCB11 and ABCB4 may be useful to determine the degree of cholestasis as well as the disease progression in PSC patients.

## Data Availability

The raw data of our nanoString experiment used to support the findings of this study are available from the corresponding author upon request.

## Supplementary Materials

Supplementary Materials Supplementary Table 1: summary table of clinical parameters in all 43 patients. Supplementary Table 2: summary table of histology parameters (Nakanuma score and stage) and expression and localization pattern of ABCB11 and ABCB4 in hepatocytes of all 43 patients. Supplementary Table 3: summary of antibodies, used in immunohistochemistry/immunofluorescence. Supplementary Table 4: summary table of patients from whom liver tissue was used for nanoString gene expression analysis. Supplementary Table 5: oligonucleotide sequences of nCounter probes for reference genes (UBC, ACTB, GAPDH, and PEX11B) and genes of interest, ABCB11, and ABCB4. Supplementary Figure 1: hematoxylin and eosin- (HE) stained slides used for LCM from liver explants with different stages of liver disease (Nakanuma 1: unremarkable liver paranchyma; Nakanuma 4: intrahepatic cholestasis, cirrhosis with formation of regenerative nodules, and bile duct proliferation). Black arrows show in low magnification, an example of areas where hepatocytes were extracted; zoomed in image shows hepatocytes in high magnification extracted for RNA extraction. Supplementary Figure 2A: immunohistochemical staining for ABCB4 and ABCB11 in two different areas of FFPE liver sections of two siblings with a homozygous mutation (p.H1238Y) in the ABCB4 gene causing PFIC3 (PFIC3, *n*=2). ABCB4 and ABCB11 stained in brown; nuclei stained with hematoxylin in blue. Inhomogeneous/patchy expression/distribution of both transporters at the bile canalicular membrane is noted in hepatocytes. Scale bars = 20 *μ*m. Supplementary Figure 2B: immunohistochemical staining for ABCB11 and ABCB4 in a PFIC1 patient, a patient with chronic hepatitis C and a patient with FNH. ABCB11 and ABCB4 stained in brown; nuclei stained with hematoxylin in blue. The PFIC1 patient showed homogeneous ABCB11 staining with cytoplasmic mislocalization and areas of accumulation at the bile canalicular membrane and inhomogeneous expression and accumulation of ABCB4 at the bile canalicular membrane. The patient with chronic hepatitis C showed inhomogeneous expression with areas of weak staining of both transporters. The FNH patient showed homogeneous expression of ABCB11 at the bile canalicular membrane and inhomogeneous ABCB4 expression with areas of ABCB4 accumulation at the bile canalicular membrane. Scale bar = 20 *μ*m. Supplementary Figure 2C: immunohistochemical staining for ABCB11 in different PSC patients. ABCB11 stained in brown; nuclei stained with hematoxylin in blue. High expression and accumulation of ABCB11 at the bile canalicular membrane of adjacent hepatocytes are noted. Scale bar = 20 *μ*m. Supplementary Figure 2D: immunohistochemical staining for ABCB4 in different PSC patients. ABCB4 stained in brown; nuclei stained with hematoxylin in blue. High expression and accumulation of ABCB4 at the bile canalicular membrane of adjacent hepatocytes are noted. Scale bars = 20 *μ*m. Supplementary Figure 3: hematoxilin and eosin (HE) staining of a normal liver and a cirrhotic liver explaining the liver architecture. Normal liver: the normal liver lobules consist of multiple acini connected with a central vein and surrounded by portal tracts, which are important for the blood supply and perfusion of the liver. Hepatocytes in liver acini are divided into 3 zones: zone 1 (periportal hepatocytes), zone 2 (hepatocytes between portal tracts and central vein area, and zone 3 (hepatocytes in the central vein area). Portal tract: (1) portal vein, (2) bile duct, and (3) hepatic artery, and (4) central vein. The cirrhotic liver has decreased number of portal tracts, forming regenerative nodules as well as showing bile duct proliferation as a sign of compensation and regeneration. Supplementary Figure 4A: PEX11B (reference gene) expression in nCounter analysis of LCM hepatocytes of FFPE liver explants from PSC patients (PSC, *n*=10), patients with non-PSC liver diseases (Other, *n*=8), and two siblings with a homozygous mutation (p.H1238Y) in the ABCB4 gene causing PFIC3 (PFIC3, *n*=2), compared with patients with non-PSC liver diseases (Other, *n*=8). Equal RNA expression of PEX11B in all 3 groups (*p* < 0.4245, PSC vs. Other; *p* < 0.2886, PFIC vs. Other). Supplementary Figure 4B: immunofluorescence analysis of PEX11B in FFPE liver explants from patient with PCLD, FNH, HepC, PSC, and two siblings with a homozygous mutation (p.H1238Y) in the ABCB4 gene causing PFIC3. PEX11B is stained in red (ALEXA 568); nuclei are stained in blue (Hoechst). Equal perinuclear expression and distribution of PEX11B-positive peroxisomes in hepatocytes. Scale bar = 10 *μ*m. Supplementary Figure 5: ABCB11 and ABCB4 expression in nCounter analysis of LCM hepatocytes of FFPE liver explants from two siblings with a homozygous mutation (p.H1238Y) in the ABCB4 gene causing PFIC3 (PFIC3, *n*=2) compared to patients with non-PSC liver diseases (Other, *n*=8). Increased RNA expression of ABCB11 (^*∗∗*^*p* < 0.0095) and ABCB4 (^*∗∗∗∗*^*p* < 0.0001) in PFIC3 hepatocytes when compared to patients with non-PSC liver diseases.

## References

[1] Stapelbroek J. M., Van Erpecum K. J., Klomp L. W. J., Houwen R. H. J. (2010). Liver disease associated with canalicular transport defects: current and future therapies. *Journal of Hepatology*.

[2] Gotthardt D., Runz H., Keitel V. (2008). A mutation in the canalicular phospholipid transporter gene, ABCB4, is associated with cholestasis, ductopenia, and cirrhosis in adults. *Hepatology*.

[3] Amer S., Hajira A. (2014). A comprehensive review of progressive familial intrahepatic cholestasis (PFIC): genetic disorders of hepatocanalicular transporters. *Gastroenterology Research*.

[4] Anzivino C., Odoardi M. R., Meschiari E. (2013). ABCB4 and ABCB11 mutations in intrahepatic cholestasis of pregnancy in an Italian population. *Digestive and Liver Disease*.

[5] Dixon P. H., Sambrotta M., Chambers J. (2017). An expanded role for heterozygous mutations of ABCB4, ABCB11, ATP8B1, ABCC2 and TJP2 in intrahepatic cholestasis of pregnancy. *Scientific Reports*.

[6] Häussinger D., Keitel V., Kubitz R. (2017). *Hepatobiliary Transport in Health and Disease*.

[7] Fickert P., Zollner G., Fuchsbichler A. (2002). Ursodeoxycholic acid aggravates bile infarcts in bile duct-ligated and *MDR2* knockout mice via disruption of cholangioles. *Gastroenterology*.

[8] Pauli-Magnus C., Kerb R., Fattinger K. (2004). BSEP andMDR3 haplotype structure in healthy Caucasians, primary biliary cirrhosis and primary sclerosing cholangitis. *Hepatology*.

[9] Carey E. J., Ali A. H., Lindor K. D. (2015). Primary biliary cirrhosis. *The Lancet*.

[10] Purohit T., Cappell M. S. (2015). Primary biliary cirrhosis: pathophysiology, clinical presentation and therapy. *World Journal of Hepatology*.

[11] Karlsen T. H., Boberg K. M. (2013). Update on primary sclerosing cholangitis. *Journal of Hepatology*.

[12] Karlsen T. H., Folseraas T., Thorburn D., Vesterhus M. (2017). Primary sclerosing cholangitis—a comprehensive review. *Journal of Hepatology*.

[13] Nakanuma Y., Harada K., Katayanagi K., Tsuneyama K., Sasaki M. (1999). Definition and pathology of primary sclerosing cholangitis. *Journal of Hepato-Biliary-Pancreatic Surgery*.

[14] Nakanuma Y., Zen Y., Harada K. (2010). Application of a new histological staging and grading system for primary biliary cirrhosis to liver biopsy specimens: interobserver agreement. *Pathology International*.

[15] de Vries E. M. G., de Krijger M., Färkkilä M. (2017). Validation of the prognostic value of histologic scoring systems in primary sclerosing cholangitis: an international cohort study. *Hepatology*.

[16] Pan J., Thoeni C., Muise A., Yeger H., Cutz E. (2016). Multilabel immunofluorescence and antigen reprobing on formalin-fixed paraffin-embedded sections: novel applications for precision pathology diagnosis. *Modern Pathology*.

[17] Longuespée R., Casadonte R., Schwamborn K. (2018). Proteomics in pathology. *Proteomics*.

[18] Baghdasaryan A., Fuchs C. D., Österreicher C. H. (2016). Inhibition of intestinal bile acid absorption improves cholestatic liver and bile duct injury in a mouse model of sclerosing cholangitis. *Journal of Hepatology*.

[19] Fuchs C. D., Paumgartner G., Wahlström A. (2017). Metabolic preconditioning protects BSEP/ABCB11−/− mice against cholestatic liver injury. *Journal of Hepatology*.

[20] Ziol M., Barbu V., Rosmorduc O. (2008). ABCB4 heterozygous gene mutations associated with fibrosing cholestatic liver disease in adults. *Gastroenterology*.

[21] Wang R., Chen H.-L., Liu L., Sheps J. A., Phillips M. J., Ling V. (2009). Compensatory role of P-glycoproteins in knockout mice lacking the bile salt export pump. *Hepatology*.

[22] Megaraj V., Iida T., Jungsuwadee P., Hofmann A. F., Vore M. (2010). Hepatobiliary disposition of 3*α*,6*α*,7*α*,12*α*-tetrahydroxy-cholanoyl taurine: a substrate for multiple canalicular transporters. *Drug Metabolism and Disposition*.

[23] Henkel A. S., Gooijert K. E. R., Havinga R., Boverhof R., Green R. M., Verkade H. J. (2013). Hepatic overexpression of ABCB11 in mice promotes the conservation of bile acids within the enterohepatic circulation. *American Journal of Physiology-Gastrointestinal and Liver Physiology*.

[24] Smit J. J. M., Schinkel A. H., Elferink R. P. J. O. (1993). Homozygous disruption of the murine *MDR2* P-glycoprotein gene leads to a complete absence of phospholipid from bile and to liver disease. *Cell*.

[25] Wang Y.-D., Chen W.-D., Wang M., Yu D., Forman B. M., Huang W. (2008). Farnesoid X receptor antagonizes nuclear factor *κ*B in hepatic inflammatory response. *Hepatology*.

