# Supplementary material for: Expression Analysis of ATP-Binding Cassette Transporters ABCB11 and ABCB4 in Primary Sclerosing Cholangitis and Variety of Pediatric and Adult Cholestatic and Noncholestatic Liver Diseases
Source: Can J Gastroenterol Hepatol. 2019 Dec 10;2019:1085717. doi: 10.1155/2019/1085717 (PMC6925824; doi:10.1155/2019/1085717)
Supplement: Supplementary Materials — Supplementary Table 1: summary table of clinical parameters in all 43 patients. Supplementary Table 2: summary table of histology parameters (Nakanuma score and stage) and expression and localization pattern of ABCB11 and ABCB4 in hepatocytes of all 43 patients. Supplementary Table 3: summary of antibodies, used in immunohistochemistry/immunofluorescence. Supplementary Table 4: summary table of patients from whom liver tissue was used for nanoString gene expression analysis. Supplementary Table 5: oligonucleotide sequences of nCounter probes for reference genes (UBC, ACTB, GAPDH, and PEX11B) and genes of interest, ABCB11, and ABCB4. Supplementary Figure 1: hematoxylin and eosin- (HE) stained slides used for LCM from liver explants with different stages of liver disease (Nakanuma 1: unremarkable liver paranchyma; Nakanuma 4: intrahepatic cholestasis, cirrhosis with formation of regenerative nodules, and bile duct proliferation). Black arrows show in low magnification, an example of areas where hepatocytes were extracted; zoomed in image shows hepatocytes in high magnification extracted for RNA extraction. Supplementary Figure 2A: immunohistochemical staining for ABCB4 and ABCB11 in two different areas of FFPE liver sections of two siblings with a homozygous mutation (p.H1238Y) in the ABCB4 gene causing PFIC3 (PFIC3, n=2). ABCB4 and ABCB11 stained in brown; nuclei stained with hematoxylin in blue. Inhomogeneous/patchy expression/distribution of both transporters at the bile canalicular membrane is noted in hepatocytes. Scale bars = 20 μm. Supplementary Figure 2B: immunohistochemical staining for ABCB11 and ABCB4 in a PFIC1 patient, a patient with chronic hepatitis C and a patient with FNH. ABCB11 and ABCB4 stained in brown; nuclei stained with hematoxylin in blue. The PFIC1 patient showed homogeneous ABCB11 staining with cytoplasmic mislocalization and areas of accumulation at the bile canalicular membrane and inhomogeneous expression and accumulation of ABCB4 at the [file 1085717.f1.docx]

**Supplementary Tables:**

**Supplementary Table 1:** Summary table of clinical parameters in all 43 patients.

**Table Legend Supplementary Table 1:**

PSC...primary sclerosing cholangitis

NC1..neonatal cholestasis related to progressive familial intrahepatic cholestasis (PFIC), NC1…neonatal cholestasis related to progressive familial intrahepatic cholestasis (PFIC), PFIC?...suspicious for PFIC, but no genetic analysis data available

NC2...neonatal cholestasis related to total parenteral nutrition (TPN)

NC3...neonatal cholestasis related to Alagille

HepC...hepatitis C

FNH..focal nodular hyperplasia

NASH...non-alcoholic-steatohepatitis

n.a...not available

**Supplementary Table 2:** Summary table of histology parameters (Nakanuma score and stage) and expression and localization pattern of ABCB11 and ABCB4 in hepatocytes of all 43 patients.

**Table Legend Supplementary Table 2:**

PSC...primary sclerosing cholangitis

NC1…neonatal cholestasis related to progressive familial intrahepatic cholestasis (PFIC), PFIC?...suspicious for PFIC, but no genetic analysis data available

NC2...neonatal cholestasis related to total parenteral nutrition (TPN)

NC3...neonatal cholestasis related to Alagille

PCLD…polycystic liver disease (non-affected, lesion free liver tissue)

HepC...hepatitis C

FNH…focal nodular hyperplasia (non-affected, lesion free liver tissue)

NASH...non-alcoholic-steatohepatitis

inh…inhomogeneous

hom…homogeneous

acc…accumulation

cm…cytoplasmic mislocalization

**Supplementary Table 3:**

Summary of antibodies, used in immunohistochemistry/immunofluorescence.

**Supplementary Table 4:**

Summary table of patients from whom liver tissue was used for nanoString gene expression analysis

**Table Legend Supplementary Table 4:**

PSC...primary sclerosing cholangitis

PFIC3…progressive familial intrahepatic cholestasis type 3 (PFIC)

PCLD…polycystic liver disease (non-affected, lesion free liver tissue)

HepC...hepatitis C

FNH…focal nodular hyperplasia (non-affected, lesion free liver tissue)

**Supplementary Table 5:**

Oligonucleotide sequences of nCounter probes for reference genes (*UBC*, *ACTB*, *GAPDH* and *PEX11B*) and genes of interest, *ABCB11* and *ABCB4*.

**Supplementary Figures:**

**Liver explant – Nakanuma Stage 1 Liver explant – Nakanuma Stage 2**

**
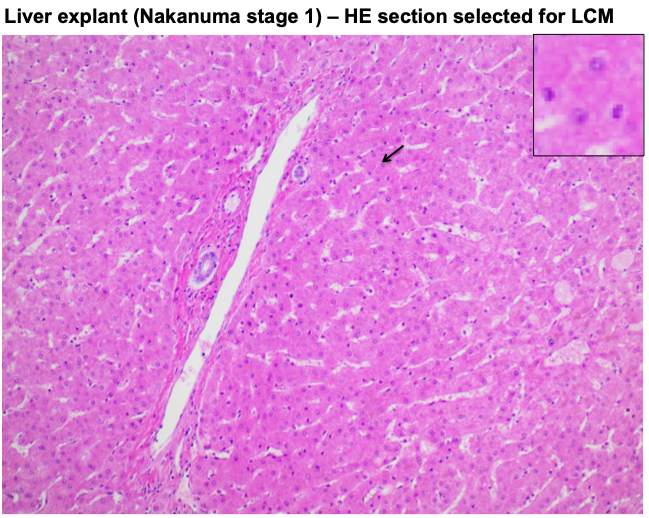

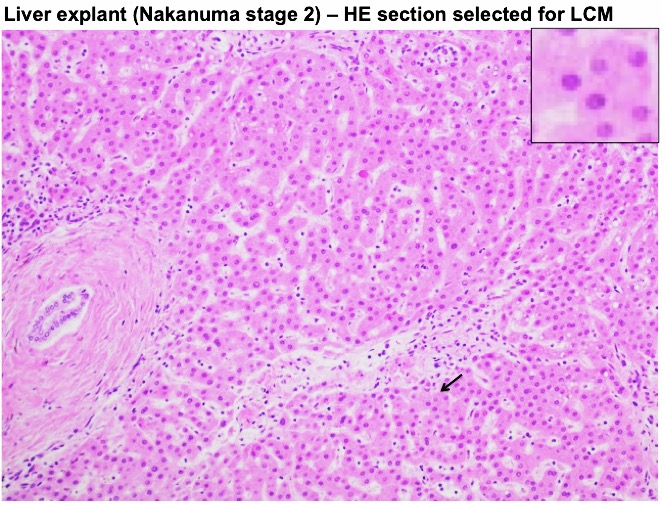
Liver explant – Nakanuma Stage 3 Liver explant – Nakanuma Stage 4**

**
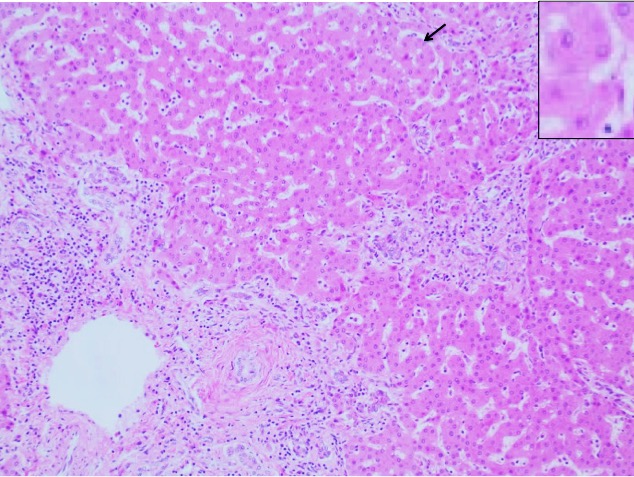

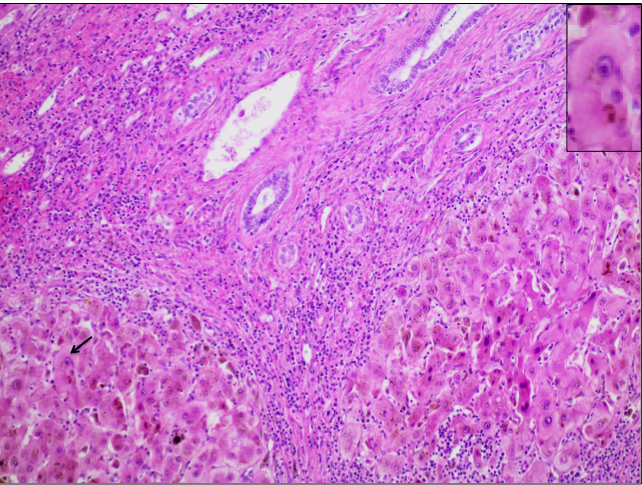
**

**Supplementary Figure 1:**

Hematoxylin and Eosin (HE) stained slides used for LCM from liver explants with different stages of liver disease (Nakanuma 1: unremarkable liver paranchyma-Nakanuma 4: intrahepatic cholestasis, cirrhosis with formation of regenerative nodules and bile duct proliferation). Black arrows showing on low magnification an example of areas where hepatocytes were extracted, zoom in images show hepatocytes in high magnification extracted for RNA extraction.


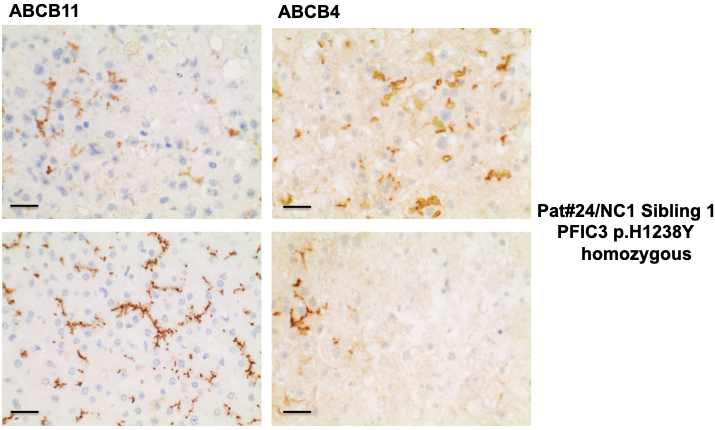


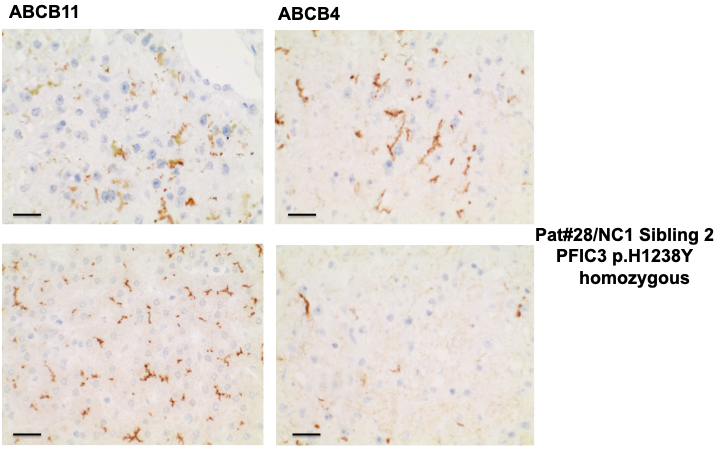


**Supplementary Figure 2A:**

Immunohistochemical staining for ABCB4 and ABCB11 in two different areas of FFPE liver sections of two siblings with a homozygous mutation (p.H1238Y) in the *ABCB4* gene causing PFIC3 (PFIC3, n=2). ABCB4 and ABCB11 stained in brown, nuclei stained with hematoxylin in blue. Inhomogeneous/patchy expression/distribution of both transporters at the bile canalicular membrane are noted in hepatocytes. Scale bars=20 µm.


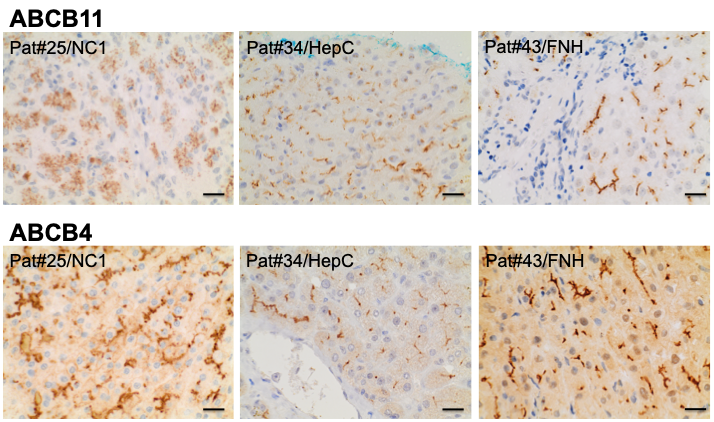


**Supplementary Figure 2B:**

Immunohistochemical staining for ABCB11 and ABCB4 in a PFIC1 patient, a patient with chronic hepatitis C and a patient with FNH. ABCB11 and ABCB4 stained in brown, nuclei stained with hematoxylin in blue. The PFIC1 patient showed homogenous ABCB11 staining with cytoplasmic mislocalization and areas of accumulation at the bile canalicular membrane and inhomogeneous expression and accumulation of ABCB4 at the bile canalicular membrane. The patient with chronic hepatitis C showed inhomogeneous expression with areas of weak staining of both transporters. The FNH patient showed homogeneous expression of ABCB11 at the bile canalicular membrane and inhomogeneous ABCB4 expression with areas of ABCB4 accumulation at the bile canalicular membrane. Scale bars=20 µm.


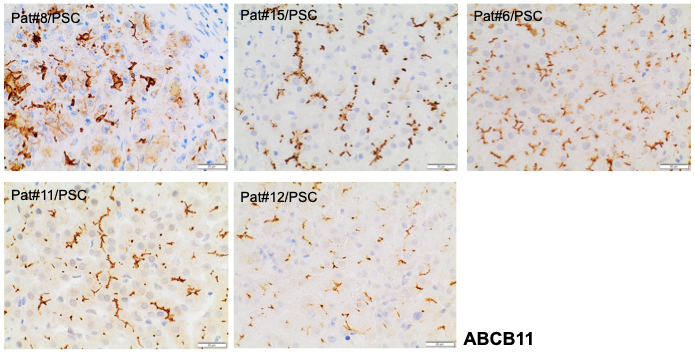


**Supplementary Figure 2C:**

Immunohistochemical staining for ABCB11 in different PSC patients. ABCB11 stained in brown, nuclei stained with hematoxylin in blue. High expression and accumulation of ABCB11 at the bile canalicular membrane of adjacent hepatocytes is noted. Scale bars=20 µm.


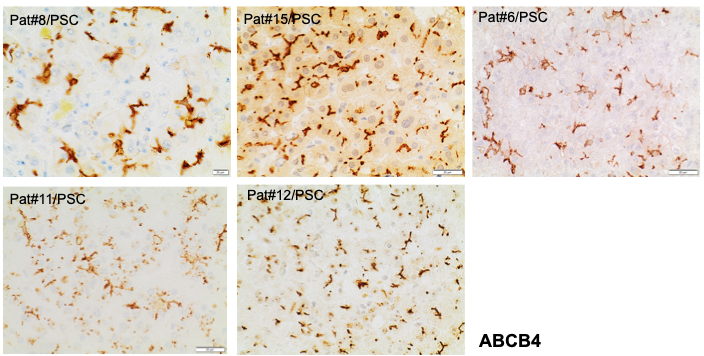


**Supplementary Figure 2D:**

Immunohistochemical staining for ABCB4 in different PSC patients. ABCB4 stained in brown, nuclei stained with hematoxylin in blue. High expression and accumulation of ABCB4 at the bile canalicular membrane of adjacent hepatocytes is noted. Scale bars=20 µm.

**
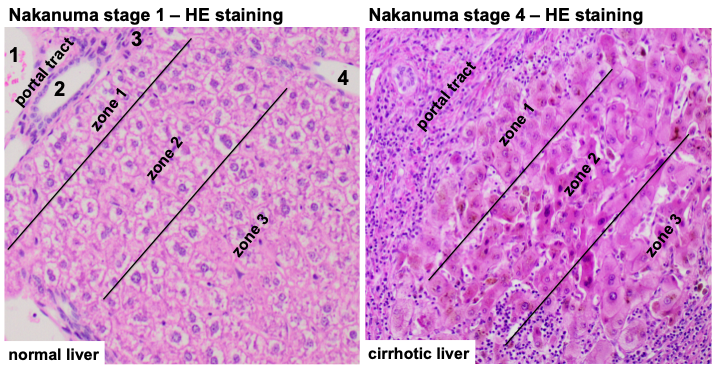
**

**Supplementary Figure 3:**

Hematoxilin and Eosin (HE) staining of a normal liver and a cirrhotic liver explaining the liver architecture. Normal liver: the normal liver lobules consists of multiple acini connected with a central vein and surrounded by portal tracts, which are important for the blood supply and perfusion of the liver. Hepatocytes in liver acini are divided into 3 zones: zone 1 (periportal hepatocytes), zone 2 (hepatocytes between portal tracts and central vein area and zone 3 (hepatocytes in the central vein area). Portal tract: (1) Portal vein, (2) bile duct, (3) hepatic artery and (4) central vein.

Cirrhotic liver has decreased number of portal tracts and forming regenerative nodules as well as show bile duct proliferation as a sign of compensation and regeneration.


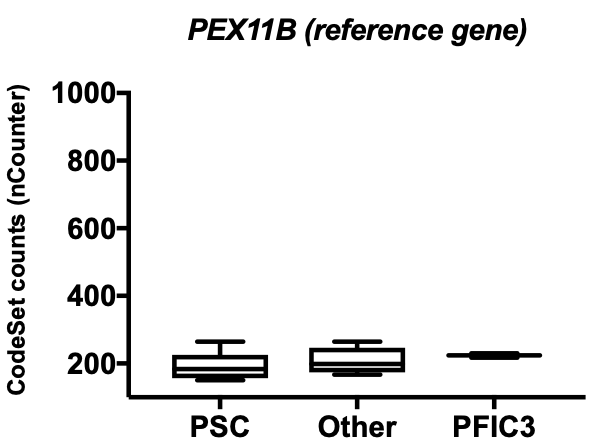


**Supplementary Figure 4A:**

*PEX11B* (reference gene) expression in nCounter analysis of LCM hepatocytes of FFPE liver explants from PSC patients (PSC, n=10), patients with non-PSC liver diseases (Other, n=8) and two siblings with a homozygous mutation (p.H1238Y) in the *ABCB4* gene causing PFIC3 (PFIC3, n=2). Equal RNA expression of PEX11B in all 3 groups (p<0.4245, PSC vs.Other, p<0.2886, PFIC vs.Other).


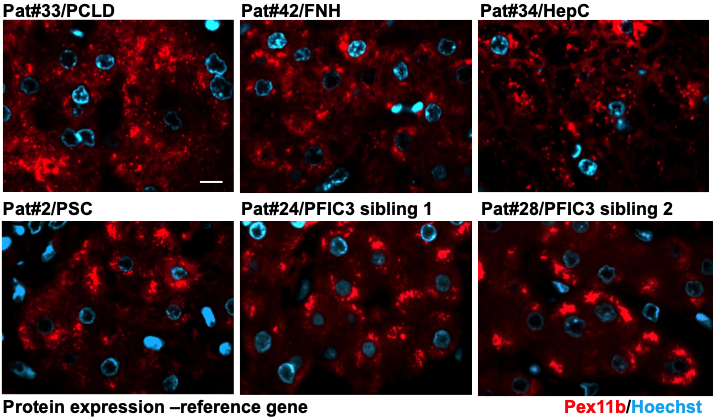


**Supplementary Figure 4B:**

Immunofluorescence analysis of PEX11B in FFPE liver explants from patient with PCLD, FNH, HepC, PSC and two siblings with a homozygous mutation (p.H1238Y) in the *ABCB4* gene causing PFIC3. PEX11B is stained in red (ALEXA 568), nuclei are stained in blue (Hoechst). Equal perinuclear expression and distribution of PEX11B positive peroxisomes in hepatocytes. Scale bar=10 µm.


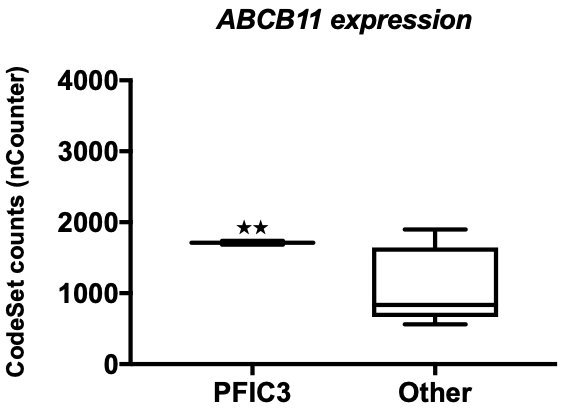

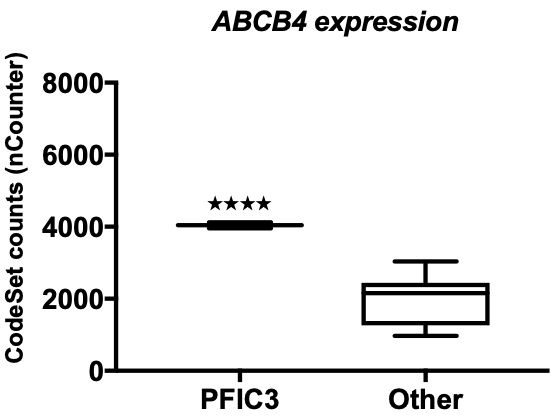


**Supplementary Figure 5:**

*ABCB11 and ABCB4* expression in nCounter analysis of LCM hepatocytes of FFPE liver explants from two siblings with a homozygous mutation (p.H1238Y) in the *ABCB4* gene causing PFIC3 (PFIC3, n=2) compared to patients with non-PSC liver diseases (Other, n=8). Increase RNA expression of *ABCB11* (^★★^p<0.0095) and *ABCB4* (^★★★★^p<0.0001) in PFIC3 hepatocytes when compared to patients with non-PSC liver diseases.
